# Survey measures of metacognitive monitoring are often false

**DOI:** 10.3758/s13428-025-02621-6

**Published:** 2025-02-18

**Authors:** Kit S. Double

**Affiliations:** University of Sydney, Sydney, Australia

**Keywords:** Metacognition, Self-awareness, Self-reports, Performance monitoring

## Abstract

Metacognitive monitoring is an extremely important ability that predicts a wide range of outcomes. However, do people have insight into their own metacognitive monitoring capacity? This study measured participants' perceived metacognitive monitoring abilities using a novel psychometrically validated questionnaire (Study 1) and then examined how well survey responses aligned with online measures of metacognitive monitoring (resolution, discrimination, sensitivity, efficiency) taken from confidence ratings participants made while performing a perceptual decision-making task and Raven’s Progressive Matrices (Study 2). We found a negative correlation between the questionnaire responses and many of the online measures of metacognitive monitoring – those who reported being better at metacognitive monitoring, in fact tended to be worse according to the online metacognitive ratings. This occurred because, in general, high self-perceptions of monitoring ability were, in fact, related to higher confidence and lower cognitive performance. These findings suggest that we may have inaccurate insights into our own metacognitive monitoring capacity and questionnaire-based measures of metacognitive abilities may be problematic as they may represent unrealistic self-perceptions.

## Introduction

Metacognitive monitoring is a vital skill. Metacognitive monitoring predicts academic performance (Nietfeld et al., 2005), decision-making (Yeung & Summerfield, 2012), and psychiatric symptoms (Rouault et al., 2018). Though defining metacognition and its constituent components is difficult, it has typically been viewed as one’s knowledge of cognition (monitoring) and regulation of cognition (Nelson & Narens, 1994). Monitoring is seen as crucial for the regulation of thought as it allows individuals to identify what they know and what they do not know (Flavell, 1979), and this monitoring facilitates various control processes such as resource allocation, strategy selection, and error detection (Nelson, 1990). Metacognitive monitoring is also inherently linked to self-awareness and consciousness (Koriat, 2007; Pasquali et al., 2010).

There are two distinct ways of measuring metacognitive monitoring (Katyal & Fleming, 2024). The first, dominant in psychology research, is through the use of online metacognitive ratings. These ratings take various forms, most notably confidence ratings (Fleming & Lau, 2014; Schraw, 2009) and judgments of learning (Double, 2023; Koriat et al., 2008). These ratings are elicited in an ‘online’ fashion, while participants are performing a cognitive task. Researchers then assess metacognitive monitoring by comparing these metacognitive judgments with actual performance, with the degree of association between accuracy and the metacognitive rating taken to be a quantitative measure of metacognition. The other dominant approach, more typical within education research, is the use of self-report questionnaires (Schraw & Dennison, 1994). There are many available survey-based measures of metacognition and they are the most popular method of measuring metacognition in educational contexts (Gascoine et al., 2017). Each of these measurement paradigms has received critique. For instance, many studies have suggested that online measures of metacognition are reactive (Double & Birney, 2017, 2019a, b; Mitchum et al., 2016; Soderstrom et al., 2015). In turn, self-report survey measures of metacognition have been criticized for not aligning with actual behavior (Veenman et al., 2006) and for lacking comprehensive reliability and validity information (Gascoine et al., 2017). In addition, survey-based measures may be more susceptible to general issues often found in questionnaire responses, such as faking and demand characteristics (Walker & MacCann, 2023).

While there are undoubtedly strengths and weaknesses of each approach, there is a more fundamental question at the heart of this distinction – can people self-report their own awareness? The hugely influential paper by Nisbett and Wilson (1977) argued that people often lack introspective awareness into their own cognitive process and often rely on inference and a priori causal theories. It would seem particularly difficult to accurately self-report metacognitive skills because metacognition requires introspection and thus one is introspecting about their ability to introspect. Katyal and Fleming (2024, p. 224) argue that survey-based measures of metacognitive capacities “are on shaky ground, precisely because self-report questionnaires presuppose the metacognitive awareness of mental function that they seek to measure”. For example, items on the metacognitions questionnaire (MCQ; Wells & Cartwright-Hatton, 2004) ask participants to rate their agreement with items like “I am constantly aware of my thinking” and “I monitor my thoughts”. To be able to accurately rate one’s agreement with such items, one must possess the quality being assessed (metacognitive ability) which is inherently problematic. This fundamental tension is obvious when considering the assumptions of the two methodological approaches. The online measurement approach defines metacognitive ability as the (lack of) *discrepancy* between one’s subjective beliefs (e.g. confidence) and reality (i.e., accuracy). In contrast, the self-report approach assumes one’s subjective beliefs accurately reflect their true (metacognitive) capabilities.

### Relationship between self-report and online measures

There is mixed evidence that survey-based measures of metacognition correlate with online rating measures of metacognition. A meta-analysis of 23 studies by Craig et al. (2020) found a small positive (*r* = .22) correlation between online and survey-based measures of metacognition. However, their analysis included a broad range of online measures (e.g., think-aloud protocols). Many studies have shown no evidence of a correlation between survey-based measures and measures derived from metacognitive ratings (Craig et al., 2020; Dörrenbächer-Ulrich & Perels, 2023; Händel & Dresel, 2022; Saraç & Karakelle, 2012; Schraw & Dennison, 1994; Terneusen et al., 2024). However, the extent to which survey-based measures align with online measures may depend on the subscales used and the components of metacognition assessed by the self-report questionnaire (Händel & Dresel, 2022).

Another limitation of the extant literature is the way metacognitive monitoring has been parametrized from online ratings, with traditional online measures being affected by cognitive performance (i.e., performance on the cognitive task on which confidence ratings are made) and biased by a subject’s average confidence. The last 15 years have seen a rapid development in our ability to parameterize metacognitive monitoring from confidence ratings (Katyal & Fleming, 2024), most notably through the use of signal detection (Fleming & Lau, 2014) and hierarchical Bayesian methods (Fleming, 2017), which have been introduced to disentangle metacognitive monitoring accuracy from cognitive performance and biases in average confidence (Fleming, 2017). To our knowledge, no studies have examined the relationship between self-reported metacognitive monitoring and these newer signal detection-based measures of metacognition.

### Current study

In the current study, we develop a novel self-report measure of metacognitive monitoring and then examine its relationship with online measures of monitoring ability. We opted to develop a new self-report measure of metacognitive monitoring because, as discussed, the existing scales have a mixture of different metacognitive processes within them, and these different components of metacognition may be distorting our understanding of the relationship between self-report and online measures of metacognition (Terneusen et al., 2024). For example, the popular *Metacognitive Awareness Inventory* (Schraw & Dennison, 1994) asks respondents questions about their learning strategies and ability to organize information. In order to address the relevant theoretical question – do self-reports of metacognitive monitoring align with online measures of monitoring? – we developed a more narrowly focused questionnaire. To ensure that our findings were not due to issues with the new questionnaire, we approached its development as we would if developing any new questionnaire by ensuring it met the normal psychometric requirements used in scale development (Study 1).

We then examine the relationship between the survey responses and online measures of metacognitive monitoring collected from participants performing a reasoning and a perceptual task (Study 2). While confidence ratings typically converge into one general factor, their calibration can differ, especially when comparing perceptual with reasoning tasks (Kleitman & Stankov, 2001). We therefore utilize two distinct cognitive tasks (perceptual and reasoning) to assess online measures of metacognition in order to capture monitoring in different contexts.

In addition, we expand on previous findings by utilizing signal detection and hierarchical Bayesian measures of metacognition. These measures allow us to parameterize metacognition in a way that better controls for performance on the cognitive task and biases in average confidence (Fleming & Lau, 2014).

### Hypotheses and data analysis

Based on the extant literature, there is reason to hypothesize that online measures will show little alignment with survey-based measures of metacognition. Existing evidence suggests there is, at best, a small positive correlation between the two types of measures (Craig et al., 2020), and there are significant distinctions in the theoretical assumptions underlying each approach (Katyal & Fleming, 2024). To examine this hypothesis, we will first analyze correlations between the survey measure developed in Study 1 and various online metacognitive measures (discrimination, resolution, sensitivity, and efficiency). We will then use regression analyses to understand how task performance and task confidence independently predict scores on our survey-based metacognition measure.

## Study 1

The aim of Study 1 was to perform a typical scale development study and produce a reliable survey measure of participants’ self-reported metacognitive monitoring ability.

### Method

#### Participants

Participants were recruited online using Prolific Academic (https://www.prolific.com). Participants were pre-screened to be residing in an English-speaking country and fluent in English. Participants were purposefully sampled for gender parity (half male participants, half female participants) and variation in age. While sample size requirements for exploratory and confirmatory factor analyses depend on various model characteristics (factor loadings, item count, and variable distributions), researchers generally recommend at least 200–300 participants to ensure reliable results (Kline, 2023; Pearson & Mundform, 2010; Tabachnick & Fidell, 2014). We aim to meet this recommendation, with 300 participants (49.67% female, M_age_ = 39.69, SD = 13.04) completing the study.

#### Materials and procedure

To develop an initial item pool, we included item content that aligned with existing survey-based measures of metacognition, e.g., *metacognitive awareness inventory* (Schraw & Dennison, 1994). Sixty-four items were included in the original item pool and are available on the Supplementary Materials. Items were selected to focus on the monitoring capacity of participants (“I evaluate how confident I am after each decision”, “I deliberately assess my performance on all tasks”) as well as aspects of monitoring related to self-awareness (“I am aware of my strengths and weaknesses”, “I know when I am performing well”).

Participants completed the items in a randomized order. Each item was rated on a Likert-like scale from 1 – “Strongly Disagree” to 6 – “Strongly Agree”. Two attention check items were intermixed into the survey (e.g., “Please select Disagree to show you are paying attention”).

### Results and discussion

One participant failed both attention check items and was removed from the analysis. We first ran an exploratory factor analysis (EFA) to determine the number of factors. An EFA with principal axis factoring and ‘promax’ rotation was performed. We selected the number of factors based on Cattell’s criterion (Cattell, 1966), in which a sharp elbow indicates when there is little benefit to retaining additional factors. Based on this criterion, a one- (28.5% of variance accounted for), two- (35.5% of variance accounted for) or three- (36.3% of variance accounted for) factor solution was viable. Given our desire for parsimony and the fact that the three-factor solution accounted for relatively little extra variance compared to a two-factor solution, we opted for a two-factor model. Factors represented an online performance monitoring factor (e.g., “When I perform tasks, I keep close track of how well I am doing) and a self-awareness factor (“I am aware of my strengths and weaknesses”). Factor loadings and the scree plot can be found in the Supplementary materials (https://osf.io/4jyxb/).

To keep the scale relatively brief and to increase the parsimony of the final scale we then selected a final eight-item version of the scale (four items for each factor). The final items were selected to maximize both factor loadings and the breadth of content coverage as well as to minimize issues with fit (i.e., modification indices indicated cross-loadings or correlated error between pairs of items). The final eight items are presented in Table 1.

**Table 1:** Items in the final metacognitive monitoring scale

Item	Factor loading (CFA)
Online monitoring	
I deliberately assess my performance on all tasks	.92
I evaluate how confident I am after each decision	.84
When I perform tasks, I keep close track of how well I am doing	.78
I always assess my performance	.86
Metacognitive Awareness	
I'm realistic about my capabilities	.56
I know when I am performing well	.72
I am aware of my strengths and weaknesses	.58
I tend to be accurate in my self-assessments	.73

We performed a confirmatory factor analysis (CFA) on this final set of eight items using the intended two-factor structure. All fit indices are within an acceptable or good range, χ^2^(19) = 50.64, CFI = .963, TLI = .946, RMSEA = .075, SRMR = .042. The factor loadings ranged from .58 to .92 and the factors were only moderately correlated covariance = .455. The total scale (Cronbach's alpha = .82) and both sub-scales showed good reliability - online monitoring: Cronbach's alpha = .83 and metacognitive awareness: Cronbach's alpha = .80.

Study 1 developed a novel scale designed to specifically target the monitoring aspects of metacognition. The final survey demonstrated good psychometric properties and the content of the items map onto both the metacognitive process of monitoring and its eventual outcome, metacognitive awareness. Using this scale, we now turn in Study 2 to examine whether survey measurements of one’s metacognitive monitoring predict the online measures typically used in psychology and neuroscience research. If participants can accurately report their metacognitive monitoring capabilities using a survey-based measure, one might expect the metacognitive awareness factor to be more positively related to online measures because it is focused on the accuracy of the metacognitive monitoring process (e.g., “I tend to be accurate in my self-assessments). In contrast, this might not apply to the same extent to the online monitoring factor, which focuses on an individual’s engagement with the process of monitoring (e.g., “I always assess my performance”) and thus an individual can respond to items in this factor affirmatively without necessarily implying that they are accurate in their monitoring.

## Study 2

Online Supplementary Materials are available on the OSF (https://osf.io/4jyxb/). Study 2 utilizes both the self-report measure of metacognitive monitoring ability developed in Study 1 and online measures of metacognition derived from confidence ratings elicited during a perceptual discrimination task and a reasoning task (Raven’s Progressive Matrices).

### Method

#### Participants

Participants were again recruited from Prolific Academic. The same screening criteria were used as Study 1. The study was run in two sessions (described below). Our desired sample size was based on a power analysis designed to detect a small correlation (*r* = .20, power = .80, α = .05), which suggested a minimum sample of 191. As attrition between the sessions was unknown, we recruited 273 participants who completed the first session. One hundred seventy-nine participants completed both sessions of the study and were used in the analysis (53% female; M_age_ 42.54, SD = 13.66). As only 87 participants provided confidence ratings on Raven’s Progressive Matrices (see Materials and procedure for details) our analysis using these data had less power to detect small effects.

#### Materials and procedure

Participants completed the measures described below online. The study was run in two sessions ~ 4 weeks apart to minimize reactivity (e.g., performance on the reasoning task influencing the self-report measure of metacognition). For practical reasons, we did not randomize the sequence of tasks. In the first session, participants completed Raven’s Progressive Matrices and the perceptual task. In the second session, participants completed the eight-item self-report measure of metacognitive monitoring developed in Study 1.

**Raven’s Progressive Matrices (RPM; **Raven & Court, 1998**):** RPM is a nonverbal assessment of a person’s reasoning and problem-solving ability (i.e., fluid intelligence). On each item, participants view a 3 x 3 matrix of abstract shapes with a missing component that they must deduce. Participants completed a computerized 12-item set drawn from the Advanced version of the task. Reduced versions of RPM have been shown to have similar concurrent validity and predictive power as the full version (Bors & Stokes, 1998). Items were ordered in the traditional fashion, which roughly corresponds to ascending difficulty. After each response, participants were prompted to rate their confidence on a six-point scale. The scale was given verbal anchors of “Guessing” and “Certain”. As eliciting such ratings has been shown to be reactive (Double, 2023; Double & Birney, 2017, 2019a), we only elicited these ratings from a subset of our sample so that we could explore whether the relationship between the self-report measure was impacted by the presence/absence of confidence ratings. As such, confidence ratings were only collected for a subset of participants (*N* = 87) on Raven’s Progressive Matrices and thus online monitoring metrics from Raven’s are confined to this subset. However, we found no evidence of reactivity and thus do not discuss this finding further here.

**Fig. 1:**
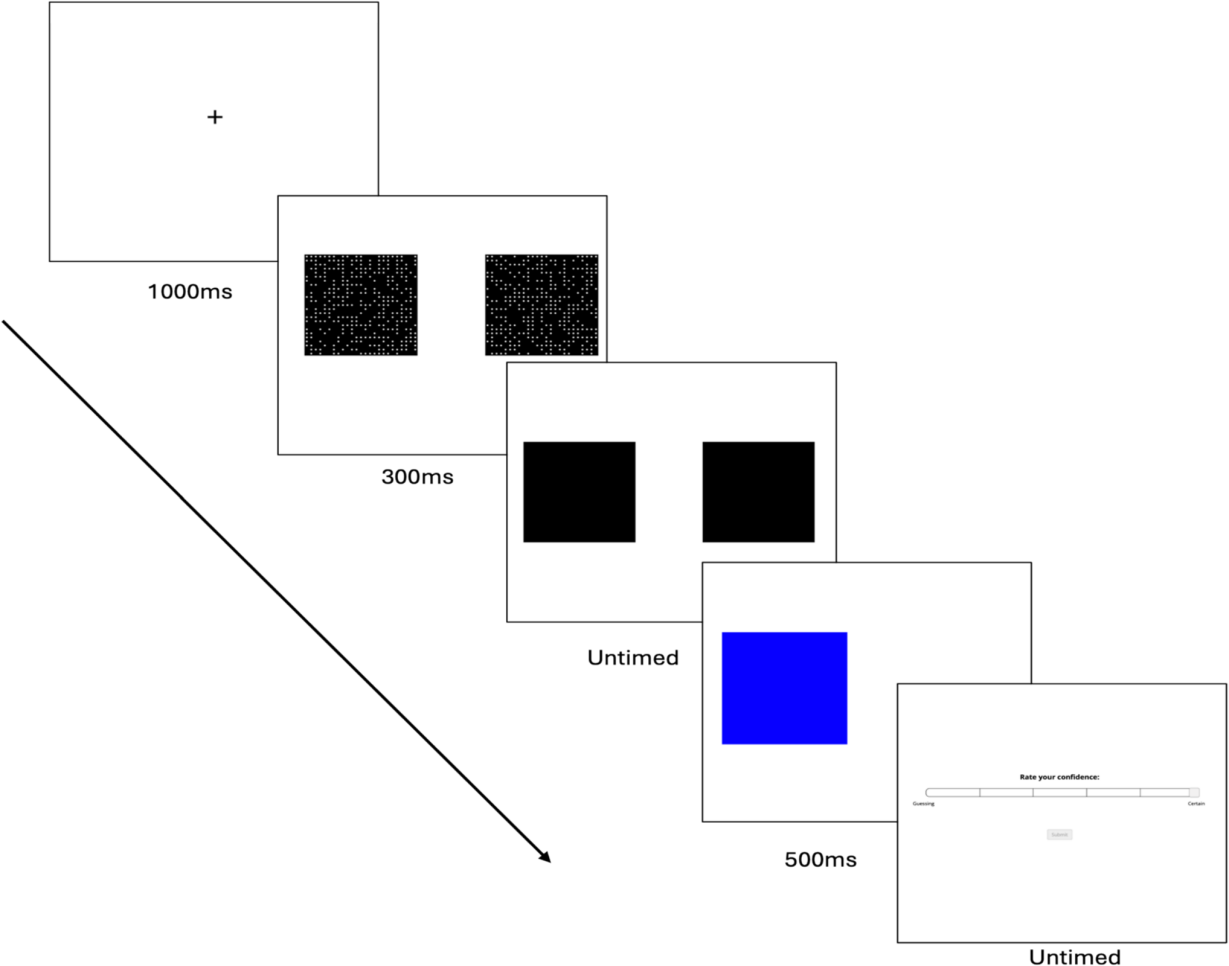
An example trial from the perceptual decision-making task used in Experiment 2

**Perceptual decision-making task:** The perceptual decision-making task was adapted from Rouault et al. (2018). The task requires participants to identify which of two boxes contains more dots, see Fig. 1. Participants completed one practice block of 16 trials and three test blocks, each with 36 trials. Each trial begins with a fixation cross for 1000 ms. Participants then saw two black boxes with randomly positioned white dots for 300 ms. One square is half-filled (313 out of a possible 625 positions), while the other contains +1–70 additional white dots. After 300 ms, the dots disappeared, and participants indicated whether they believed the left or right box had more dots (using the ‘w’ and ‘e’ keys, respectively). Participants had unlimited time to make their decision. As the signal detection measures of metacognition require a constant level of difficulty (Fleming & Lau, 2014), we used a two-down, one-up staircasing procedure to maintain a constant level of performance during the experiment and across participants. Equal step sizes for steps up and down were used. Staircasing began in the practice block, with step size calculated in log space, with a starting point of 4.2 (+70 dots), changing by ±0.4 for the first five trials, ± 0.2 for the next five trials and ± 0.1 for the rest of the task.

After participants responded, their response was confirmed visually by coloring their selected box blue for 500 ms. All participants then made a confidence rating. Participants were asked to “Rate their Confidence” using their mouse along a six-point slider ranging from ‘Guessing’ to ‘Certain’. Confidence ratings were untimed. Feedback was provided only in the practice block.

**Metacognitive ability self-report:** The eight-item final version of the survey developed in Study 1 was administered. Items were presented in random order with the same ‘Strongly Agree’ to ‘Strongly Disagree’ scale used. We computed scores for each of the subscales separately as well as a total score across all eight items.

#### Online measures of metacognition

Many different online measures of metacognition exist, each with their own strengths and weaknesses (for a review, see Fleming & Lau, 2014). We use a wide range of measures across two distinct types of tasks (perceptual and higher-order reasoning) to better capture metacognitive monitoring.

**Resolution:** Resolution is a measure of the relationship (correlation) between confidence and performance across the task (Maki et al., 2005). It is typically measured using an intra-individual correlation between confidence and accuracy. A variety of correlational measures have been used (Pearson’s *r*, Goodman–Kruskal gamma correlation, the phi correlation) as well as signal detection measures (see below). We used the Goodman–Kruskal gamma correlation as our measure of resolution. Stronger positive correlations are observed when a person can accurately judge the likelihood of correct performance relative to others.

**Discrimination:** Discrimination measures the degree to which a person’s confidence differs between correct and incorrect responses (Schraw, 2009). It is computed as the difference between average confidence on correct versus incorrect responses. A positive value indicates a person was more confident when correct than incorrect (i.e., metacognitive awareness).

**Metacognitive sensitivity:** The approaches outlined above may provide more biased estimates of metacognition compared to measures based on signal detection theory (Maniscalco & Lau, 2012). An alternative, metacognitive sensitivity, is measured by fitting a generative model of confidence using a signal detection model to two-alternative forced-choice tasks. This approach parameterizes metacognitive sensitivity as the extent to which a person’s confidence distinguished between correct and incorrect items, known as meta-d’ (Maniscalco & Lau, 2012). Here, we use a Hierarchical Bayesian estimation procedure of meta-d’, which is outlined in Fleming (2017). Note that estimating metacognitive sensitivity requires several assumptions, notably two alternative responses and a consistent signal strength (difficulty), thus we only calculated this measure for the perceptual discrimination task, not Raven’s Progressive Matrices.

**Metacognitive efficiency:** While metacognitive sensitivity measures how well confidence ratings discriminate correct from incorrect responses, metacognitive efficiency concerns how well confidence ratings discriminate correct from incorrect responses, relative to how informative we might expect those confidence ratings to be given the observer’s perceptual performance (Maniscalco & Lau, 2015). meta-d’ is the value of perceptual accuracy (d’) that would have been predicted to give rise to the observed confidence rating data assuming an ideal observer (Fleming, 2017). If the observer is metacognitively optimal, meta-dʹ = dʹ is expected. Metacognitive efficiency takes advantage of the ratio scaling properties of d’ measures as it is parametrized as the ratio of metacognitive sensitivity to perceptual accuracy: meta-d’/d’.

**Confidence:** In addition to the monitoring measures, we measured participants’ average confidence across the confidence ratings for each task.

### Results and discussion

#### Scale reliability

The total score on the eight-item self-report measure had good-to-excellent reliability (α = .86). Both the online monitoring (α = .88) and metacognitive awareness (α = .77) sub-scales also demonstrated good reliability. For an analysis and discussion comparing the performance of the signal detection and traditional online measures of metacognition see the Supplementary Materials.

#### Correlations

Bivariate correlations are shown in Table 2. The metacognitive monitoring subscale and the metacognitive awareness subscale were moderately positively associated with each other. In general, the survey-based measures of metacognition were *negative* predictors of metacognitive monitoring. Specifically, the self-report total and monitoring subscale negatively predicted resolution and discrimination on Raven’s Progressive Matrices. Furthermore, self-report total and online monitoring scales were negatively associated with resolution on the perceptual decision-making task, additionally, online monitoring negatively predicted discrimination on the perceptual discrimination task. The signal detection-based measures of metacognition (sensitivity and efficiency) did not show any relationship with the self-report scales.

**Table 2:** Bivariate correlations from Study 1

Variable	M (SD)	1	2	3	4	5	6	7	8	9	10	11	12
Self-report													
1. Total	37.11 (5.61)	1											
2. Monit.	17.65 (3.86)	.924***	1										
3. Awareness	19.47 (2.52)	.812***	.528***	1									
Perceptual task													
4. Perf.	0.8 (0.26)	-.151*	-.127	-.142	1								
5. Resolution	0.24 (0.22)	-.154*	-.153*	-.11	.101	1							
6. Disc.	0.32 (0.31)	-.142	-.148*	-.089	.084	.726***	1						
7. Sensitivity	0.48 (0.42)	-.092	-.088	-.071	.154*	.771***	.831***	1					
8. Efficiency	0.62 (0.72)	-.111	-.109	-.08	-.08	.688***	.651***	.767***	1				
9. Confidence	3.82 (0.93)	.304***	.295***	.227**	.017	-.216**	-.12	.208**	.022	1			
Raven's Progressive Matrices													
1. Perf.	0.37 (0.24)	-.227**	-.258***	-.111	.078	.048	.035	-.033	-.02	-.223**	1		
11. Resolution	0.5 (0.57)	-.231*	-.241*	-.13	-.004	.13	.054	.018	.152	-.237*	.339**	1	
12. Disc.	1.16 (1.32)	-.283**	-.319**	-.124	.081	-.045	.151	-.004	-.003	-.216*	.468***	.773***	1
13. Confidence	3.64 (1.06)	.103	.026	.182	-.231*	-.290**	-.265*	-.19	-.260*	.314**	.480***	.08	.054

* *p* < .05, **p < .01,*** *p* < .001. Monit. = monitoring; perf. = performance; disc. Discrimination. Note: Confidence ratings were only collected for a subset of participants (*N* = 87) on Raven’s Progressive Matrices. Relative accuracy could not be computed for 2 participants on the perceptual discrimination task and 1 participant on Raven's. Discrimination could not be computed for 1 participant on Raven's.

Furthermore, the total score and both subscales positively predicted confidence in the perceptual discrimination task. The total score and awareness scale were positively associated with confidence in Raven’s Progressive Matrices, though neither analysis reached conventional significance (though note these analyses were based on the reduced sample size who completed confidence ratings on Raven’s Progressive Matrices). Somewhat surprisingly, the self-report total and monitoring subscale were also *negatively* associated with performance on Raven’s Progressive Matrices and the total score was also negatively associated with performance on the perceptual discrimination task.

#### Confidence and accuracy models

Given that we observed relationships between the survey-based measures and both confidence and performance, we performed a series of regressions to examine the unique contribution of confidence and performance to each self-report scale. The models are presented in Table 3.

**Table 3:** Experiment 2 model results predicting self-reported metacognitive monitoring with performance and confidence on the cognitive tasks

	Total		Monitoring		Awareness		Total		Monitoring		Awareness	
	β	*P*	β	*P*	β	*P*	β	*P*	β	*P*	β	*P*
Perceptual Performance	-.16	.030	- .13	.069	- .15	.048						
Perceptual Confidence	.31	< .001	.30	< .001	.23	.002						
Reasoning Performance							-.32	.008	- .35	.004	- .17	.168
Reasoning Confidence							.26	.033	.19	.109	.26	.033
Observations	178		178		178		87		87		87	
*R*^2^	0.117		0.104		0.072		0.091		0.093		0.055	

After controlling for performance, average confidence on Raven’s Progressive Matrices was a significant positive predictor of the self-report total score and the awareness subscale. Confidence also positively predicted the monitoring scale, though it did not reach significance (possibly due to a lack of power). Controlling for average confidence, average performance was a significant negative predictor of the total scale and the monitoring subscale.

The total self-report scale score was uniquely negatively predicted by perceptual accuracy and positively predicted by average confidence of the perceptual task. Average accuracy on the perceptual task was a significant negative predictor of monitoring scale, controlling for confidence, but confidence did not quite reach conventional significance when predicting the monitoring scale when controlling for accuracy. Finally, average confidence on the perceptual task was a significant positive predictor of awareness scale, controlling for accuracy, and average accuracy was a significant negative predictor of the awareness scale when controlling for confidence. For completeness, we performed the same models with each of the online monitoring measures as the criterion variables (noting that these metrics are derived from the same data as the performance scores). These models are available in the OSF repository.

These results indicate that the survey-based measures of monitoring ability were misaligned with the online measures of metacognition. The self-report scales were generally associated with higher confidence and lower performance, as well as poorer metacognitive accuracy. Notably, our effects did not simply indicate no relationship between the self-report and online measures (which, in itself, would be a concern), we found significant moderate *negative *relationships between self-report questionnaires and online measures of metacognition, indicating that the self-reports of metacognition were reliably false. We discuss the implications this has for measuring metacognition further in the General discussion.

## General discussion

The studies presented here evaluated the relationship between a self-report questionnaire measure of metacognitive monitoring and online performance measures of metacognitive monitoring. We first developed a reliable self-report measure of metacognitive monitoring and then evaluated its relationship with various online measures of metacognitive monitoring extracted from confidence ratings made during a perceptual and a reasoning task. The results suggest that self-reports of metacognitive monitoring abilities are negatively associated with performance on both tasks and positively associated with confidence on the perceptual task. Unsurprisingly, this produced a general trend whereby self-reports of metacognitive monitoring were negatively associated with the measures of monitoring ability derived from the online confidence ratings. This finding suggests that participants’ self-perceptions of their monitoring ability were reliably inaccurate. Those that thought they were the most capable at monitoring their cognition tended to in fact to be the least able to gauge their performance. This finding calls into question the validity of using survey-based measures of metacognition.

Evidence suggests that we possess relatively little insight into our higher-order cognitive processes (Nisbett & Wilson, 1977). By its very nature metacognition is inextricably linked to awareness and consciousness (Koriat, 2006; Nelson, 1996). Given this, there is perhaps an obvious contradiction in asking people to self-report their own self-awareness, yet this is often what is expected when we ask participants to self-report the monitoring aspects of their metacognition. Our results lend support to this challenge of survey-based measures of metacognition and lay bare the inherent contradiction of having participants self-report their metacognitive capacities. We found no evidence that survey measures of metacognition aligned positively with online measures, indeed we found several online measures of metacognition were actually negatively related with survey-based measures of metacognition. We propose that measuring metacognition using self-report questionnaires is both theoretically shaky and empirically problematic and would discourage the use of such questionnaires.

Despite the theoretical similarities between metacognition and monitoring judgments, research consistently shows that survey-based metacognition measures do not align with online measures. Our study adds to this evidence, joining several others that found no correlation between these two measurement approaches (Craig et al., 2020; Dörrenbächer-Ulrich & Perels, 2023; Händel & Dresel, 2022; Saraç & Karakelle, 2012; Schraw & Dennison, 1994; Terneusen et al., 2024). This disconnect is concerning because both measurement types are widely used in metacognition research. Without agreement between our measurement tools, we cannot make firm conclusions about metacognition and risk confusing different concepts or using different terms for the same concept (jingle-jangle fallacies). As suggested by Händel and Dresel (2022), we need an integrated approach to studying metacognition. Moreover, as Katyal and Fleming (2024) argue, we need better measurement methods that maintain scientific rigor without oversimplifying this complex concept.

Interestingly, we found a negative relationship between our performance measures on both the perceptual and reasoning tasks with survey-based measures of metacognitive monitoring. A previous meta-analysis by Ohtani and Hisasaka (2018) found a small positive correlation between questionnaire measures of metacognition and intelligence (*r* = .13) and academic achievement (*r* =.19). However, it is worth noting that metacognition questionnaires often have many facets assessing learning strategies and cognitive regulation which may account for the positive relationship (and is why we developed a novel questionnaire in the current study). Historically, the evidence for a relationship between online measures of metacognition and performance is more complex as the online confidence ratings are collected during the cognitive task leading to confounds between confidence and performance (Maniscalco & Lau, 2012).

While we could offer speculation as to why self-reported metacognitive monitoring was negatively correlated with performance, this is obviously hampered by the fundamental point we are making – we do not know what it is we are measuring when we ask people to self-report their metacognition. If one assumes that participants are providing accurate responses to the questionnaire, then it might point to a dual-task cost whereby more resources spent monitoring one’s cognition affords less resources for cognitive processes and reduces performance. Evidence from reactivity research, which investigates how people perform on cognitive tasks when they are explicitly required to monitor their performance (compared to controls who are not required to do so) supports this notion. Reactivity studies have shown that the requirement to monitor directs resources to metacognitive cues, which can come at a cost to cognitive performance (Double, 2023; Double & Birney, 2017, 2019a, 2024), although, in contrast, if people are spending additional resources attending to metacognitive cues that are cognitively relevant then performance may improve (Double et al., 2018; Mitchum et al., 2016; Soderstrom et al., 2015). However, we do not believe it is a reasonable assumption to take responses to the metacognitive questionnaire at face value; a high response might actually indicate a lack of metacognition due to overconfidence (see our next point) or an ignorance of one’s ignorance as one might expect to arise based on phenomena such as the Dunning–Kruger effect (Dunning, 2011).

The current findings provide some support for the notion that self-reports of metacognitive ability are related to confidence in one’s cognitive ability, though this was significant in the case of the perceptual task not the reasoning task (which was based on analysis of the reduced sample who provided confidence ratings on the reasoning task). Self-reports of ability are conceptually and empirically similar to the notion of confidence as individuals appear to form global beliefs about their competence based on local confidence (Rouault et al., 2019). Our findings are of course somewhat different in that we found a positive relationship between self-reports of metacognitive ability with confidence in perceptual ability. This may point to the fact that confidence in one’s metacognitive abilities and cognitive abilities are related as one would expect based on the idea that confidence is somewhat domain-general (Kleitman & Stankov, 2007; Mazancieux et al., 2020; Stankov et al., 2015). Obviously, given that this relationship did not reach statistical significance when analyzing the relationship between metacognitive questionnaire responses and confidence on the reasoning task this finding should be interpreted cautiously and requires further replication.

The current findings support the notion that there is little convergent validity between survey-based measures of metacognitive monitoring and online measures of monitoring. However, it is worth noting that while we took care to develop a questionnaire for the current study that would meet conventional psychometric requirements, it does not necessarily mean that our findings would generalize to each of the questionnaire measures of metacognition used in the literature. We opted to develop a new questionnaire because the questionnaires frequently used in the literature adopt a multifaceted view of metacognition (as is typical in educational psychology) and often assess subjects’ perceptions of a range of behaviors such as study strategies and planning activities, and even cognitive capacities (for more discussion, see critiques in Katyal & Fleming, 2024). While we were able to develop a more conceptually pure measure here to illustrate our point, it is important to generalize this to other measures of metacognition cautiously. Furthermore, our study was limited to confidence ratings as the sole online measure of metacognition and included only two cognitive tasks. Therefore, these results may not extend to other types of metacognitive judgments or cognitive tasks.

Furthermore, the broad scope of our survey-based metacognition measure may limit our findings' generalizability. While our online metacognitive measures were task-specific (focusing on individual items), the survey assessed general metacognitive tendencies (e.g., "I deliberately assess my performance on all tasks"). Given that components of metacognition can be domain-specific (Alexander et al., 1995; Bellon et al., 2020; Greene et al., 2015; Händel et al., 2023; Kleitman & Stankov, 2001; Rovers et al., 2019; Veenman et al., 1997), this mismatch between specific and general measures may be problematic (Azevedo, 2009; Dinsmore et al., 2008; Rovers et al., 2019). When completing the survey, participants might have reflected on their metacognitive experiences during academic or work activities rather than the type of cognitive tasks used in our study. Future research should consider using survey measures that specifically target metacognition in the same domain as the online monitoring tasks.

The current studies examined the relationship between self-report and online measures of metacognitive monitoring. Building off recent conceptual critiques about the lack of overlap of these measures (Katyal & Fleming, 2024), we show empirically that there is no evidence of a positive relationship between these two different ways of measuring metacognition. Indeed, our results suggest they may actually be negatively related due to survey-based measures of metacognition predicting poorer cognitive performance and, at times, higher confidence. This suggests that these alternative approaches may measure two things that bear little empirical correspondence.

## Data Availability

Materials and data are available at (https://osf.io/4jyxb/). None of the reported studies were preregistered.

## Code availability

Materials and analysis code are available at (https://osf.io/4jyxb/).
